# Association of circulating angiotensin converting enzyme activity with respiratory muscle function in infants

**DOI:** 10.1186/1465-9921-11-57

**Published:** 2010-05-12

**Authors:** Gabriel Dimitriou, Despina Papakonstantinou, Eleana F Stavrou, Sotirios Tzifas, Aggeliki Vervenioti, Anny Onufriou, Aglaia Athanassiadou, Stefanos Mantagos

**Affiliations:** 1 Neonatal Intensive Care Unit, Department of Pediatrics, University of Patras Medical School, Rio, Patras, Greece; 2 Department of General Biology, University of Patras Medical School, Rio, Patras, Greece; 3 Department of Biochemistry, University Hospital of Patras, Rio, Patras, Greece

## Abstract

**Background:**

Angiotensin converting enzyme (ACE) gene contains a polymorphism, consisting of either the presence (I) or absence (D) of a 287 base pair fragment. Deletion (D) is associated with increased circulating ACE (cACE) activity. It has been suggested that the D-allele of ACE genotype is associated with power-oriented performance and that cACE activity is correlated with muscle strength. Respiratory muscle function may be similarly influenced. Respiratory muscle strength in infants can be assessed specifically by measurement of the maximum inspiratory pressure during crying (Pi_max_). Pressure-time index of the respiratory muscles (PTImus) is a non-invasive method, which assesses the load to capacity ratio of the respiratory muscles.

The objective of this study was to determine whether increased cACE activity in infants could be related to greater respiratory muscle strength and to investigate the potential association of cACE with PTImus measurements as well as the association of ACE genotypes with cACE activity and respiratory muscle strength in this population.

**Methods:**

Serum ACE activity was assayed by using a UV-kinetic method. ACE genotyping was performed by polymerase chain reaction amplification, using DNA from peripheral blood. PTImus was calculated as (Pi_mean_/Pi_max_) × (Ti/Ttot), where Pi_mean _was the mean inspiratory pressure estimated from airway pressure, generated 100 milliseconds after an occlusion (P_0.1_), Pi_max _was the maximum inspiratory pressure and Ti/Ttot was the ratio of the inspiratory time to the total respiratory cycle time. Pi_max _was the largest pressure generated during brief airway occlusions performed at the end of a spontaneous crying effort.

**Results:**

A hundred and ten infants were studied. Infants with D/D genotype had significantly higher serum ACE activity than infants with I/I or I/D genotypes. cACE activity was significantly related to Pi_max _and inversely related to PTImus. No association between ACE genotypes and Pdi_max _measurements was found.

**Conclusions:**

These results suggest that a relation in cACE activity and respiratory muscle function may exist in infants. In addition, an association between ACE genotypes and cACE activity, but not respiratory muscle strength, was demonstrated.

## Background

Angiotensin I-converting enzyme (ACE) is a zink metallopeptidase whose main functions are to convert angiotensin I into vasoactive and aldosterone-stimulating peptide angiotensin II and to degrade vasodilator kinins. Circulating ACE (cACE) is found in biological fluids and originates from endothelial cells. ACE is also an important component of the local renin-angiotensin systems (RASs), which have been identified in diverse tissues, including lung and skeletal muscles [1,2]. A polymorphism of the human ACE gene has been identified in humans and contains a polymorphism consisting of either the presence (insertion, I) or absence (deletion, D) of a 287 base pair (bp) fragment [3]. The deletion is associated with increased ACE activity in both tissue [4] and circulation [5]. Circulating ACE activity was stable when serially measured in the same individuals, while large differences among subjects were observed [6]. The I/D polymorphism accounts for approximately half of the observed variance in ACE levels [5]. However, the presence of quantitative trait loci controlling ACE levels was suggested [7].

D-allele of ACE genotype has been associated with power-oriented performance, being found in excess in short-distance swimmers [8] and with greater strength gains in the quadriceps muscle [9]. Furthermore, it has been suggested that cACE activity has been associated directly with muscle strength in healthy Caucasians, naïve to strength training [10]. Thus, respiratory muscle function and specific respiratory muscle strength may be similarly influenced.

Respiratory muscle strength in infants can be assessed specifically by measurement of the maximum inspiratory pressure during crying (Pi_max_) [11,12]. Pressure-time index of the respiratory muscles (PTImus) is a non-invasive method, which assess the load to capacity ratio of the respiratory muscles [13]. PTImus has been validated in both adults [14] and infants [15].

The aim of this study was to test the hypothesis that increased cACE activity in infants could be related to greater respiratory muscle strength assessed by measurement of Pi_max_. We further investigated the potential association of cACE with PTImus measurements, as well as the association of ACE genotypes with cACE activity and respiratory muscle strength in this population.

## Methods

### Patients

Infants cared for at the Neonatal Intensive Care Unit-Pediatric Department of the University General Hospital of Patras, Greece, were eligible for the study. Infants were entered into the study if parents gave informed written consent. The study was approved by the local Research Ethics Committee. The studied population was recruited from a study examining the association of ACE genotypes on respiratory muscle function in infants. All infants were studied before discharge, in supine position, at least one hour after a feed. Infants had no respiratory symptoms for at least 3 days before measurement. Furthermore, infants were on full oral feeds, had serum electrolytes, calcium, magnesium and phosphates within normal range and did not receive any methylxanthines. Blood sampling for circulating ACE activity determination was performed the previous or the same day of the measurements.

### **ACE genotype determination**

ACE genotyping was performed on DNA extracted from 0.5 ml of whole blood, collected from an indwelling catheter or via peripheral venipuncture during routine blood sampling. The blood samples were stored at -80°C in EDTA vacutainer tubes. The method has been previously described [16]. Briefly, DNA was extracted by using Qiamp spin columns (Blood mini kit- Qiagen, QIAGEN Inc., Germantown, U.S.A). DNA was analyzed by electrophoresis on an agarose gel. DNA amplification of the 16^th ^ACE intron was performed using two sets of primers flanking the polymorphic site, (outer and inner primers), as mistyping of the D/D genotype has been reported to occur using conventional amplification with insertion/deletion (I/D) flanking primer [17].

### **Plasma ACE activity determination**

An additional 1 ml of whole blood was collected during routine blood sampling. Serum was separated immediately from the whole blood by centrifugation at 1500 g for 10 min. The samples were stored at -20°C in vacutainer tubes until analysis. Serum ACE activity was assayed by using a UV-kinetic method (Medicon SA) and an AU480 Clinical Chemistry System (Beckman Coulter, Inc, High Wycombe, UK). The determination of ACE was based on the calculation of the rate of absorbance change at 340 nm during the hydrolysis of the substrate N-(3-(2-(furyl)acryloyl)-L-phenylalanylglycylglycine (FAPGG) to N-(3-(2-(furyl)acryloyl)-L-phenylalanine (FAP) and glycylglycine. The method has a detection limit of 7 U/L, linearity between 7-140 U/L of ACE and an intra-assay coefficient of variation between 2.26% and 4.86%.

### **Measurement of respiratory muscle function**

Airway flow was measured using a pneumotachograph (Mercury F10L, GM Instruments, Kilwinning, Scotland) connected to a differential pressure transducer (DP45, range ± 2 cm H_2_O, Validyne Corp, Northridge, CA, USA). Airway pressure (Paw) was measured from a side port on the pneumotachograph, using a differential pressure transducer (DP45, range ± 100 cm H_2_O, Validyne Corp, Northridge, CA, USA). The signals from the differential pressure transducers were amplified, using a carrier amplifier (Validyne CD 280, Validyne Corp, Northridge, CA, USA) and they were recorded and displayed in real time on a computer (Dell Optiplex GX620, Dell Inc., Texas, U.S.A) running Labview™ software (National Instruments, Austin, Texas, U.S.A) with analog-to-digital sampling at 100 Hz (16-bit NI PCI-6036E, National Instruments, Austin, Texas, U.S.A).

#### Measurement of Pi_max_

To measure Pi_max_, a facemask (total deadspace, 4.5 mL) was held firmly over the infant's nose and mouth. A small needle leak in the mask was used in order to prevent glottic closure and artificially high Pi_max _[13]. The airway was occluded at the end of a spontaneous crying effort using a unidirectional valve attached to the pneumotachograph, which allowed expiration but not inspiration. The occlusion was maintained for at least four inspiratory efforts. At least three sets of airway occlusions were performed and the maximum Pi_max _achieved for individual was recorded.

#### Measurement of PTImus

P_0.1 _was calculated as the airway pressure generated 100 milliseconds after an occlusion, while the infant was quietly breathing. At least four airway occlusions were performed and average P_0.1 _was calculated. Pressure-time index of the inspiratory muscles (PTImus), was calculated as: PTImus = (Pi_mean_/Pi_max_) × (Ti/Ttot) where Pi_mean _was the average airway pressure during inspiration, obtained from the formula Pi_mean _= 5 × P_0.1 _× Ti [18]. Pi_max _was the maximum inspiratory airway pressure, Ti was the inspiration time and Ttot was the total time for each breath, calculated from the airway flow signal.

Muscle mass increases with maturity and body growth [19] and P_imax _continues to increase outside the neonatal period [11]. Therefore, in order to examine the association of ACE genotype with respiratory muscle strength, P_imax _was also related to body weigth at the time of measurement.

### Statistical analysis

Data was tested for normality using the Shapiro-Wilk and D'Agostino skewness tests. Differences between ACE genotype groups were assessed for statistical significance, using the Kruskal-Wallis and Dunn's post-hoc non parametric and Cramer's V tests, as appropriate. Simple regression analysis was performed to determine whether cACE is related to Pi_max _and PTImus measurements. Stepwise multiple regression analysis was performed to determine if cACE activity is related to respiratory muscle strength, assessed by measurement of Pi_max _and PTImus measurements, independently to weight at measurement, ACE genotyping, postmenstrual age (PMA), gender and support from mechanical ventilation.

Statistical analysis was performed using StatView 5.0 (SAS Institute, Inc., NC, USA) and NCSS 2007 (NCSS, Utah, USA)

### Sample size

Interim analysis of the data of 50 infants demonstrated a correlation of magnitude r = 0.23 between Pi_max _and cACE activity approaching statistical significance. Recruitment of 106 subjects would allow us to detect a correlation of magnitude r = 0.24 between Pi_max _and cACE levels with 80% power at 5% significance level ("Alpha", the probability of rejecting a true null hypothesis).

## Results

### Whole study population

Between February 2007 and September 2008 one hundred ten infants were recruited. Fifty infants (45.5%) required ventilation in the initial stage of their illness with a median duration of ventilatory support of 3.3 days (range 1.5-59). The characteristics of the study population are presented in table 1.

**Table 1:** Characteristics of the whole study population

Number of studied infants	110	
Gestational age (weeks)	36	(27-40)

Birth weight (g)	2705	(960-4150)

Gender (male)	62	(56.4)

Preterm	75	(57.7)

Duration of mechanical ventilation (days)	0	(0-59)

**Diagnoses on admission**		

Respiratory distress syndrome (RDS)	37	(33.6)

Transient tachypnea of the newborn (TTN)	17	(15.5)

Meconium aspiration syndrome	5	(4.5)

Infection	16	(14.5)

Congenital pneumonia	6	(5.5)

Prematurity (gestational age ≤ 34 weeks and birthweight < 2 kg without additional problems on admission)	15	(13.7)

Birth depression	6	(5.5)

Intrauterine growth retardation	5	(4.5)

Airleaks	2	(1.8)

Meconium plug	1	(0.9)

**At the time of measurement**		

Postnatal age (days)	9	(1-107)

Postmenstrual age (PMA) (weeks)	37.1	(32.4-46.7)

Preterm (PMA<37 weeks)	57	(43.8)

Weight (g)	2633	(1850-4050)

cACE activity (U/L)	42.5	(16.7-89.0)

Pi_max _(cmH_2_O)	65.0	(36.9-91.8)

Pi_max_/weight (cmH_2_O/kg)	24.5	(14.5-47.1)

PTImus	0.062	(0.023-0.149)

Data are demonstrated as n (%) or median (range).

**Abberviations: **cACE: circulating ACE; Pi_max_: maximum inspiratory pressure during crying; PTImus: pressure-time index of the respiratory muscles

**Figure 1:**
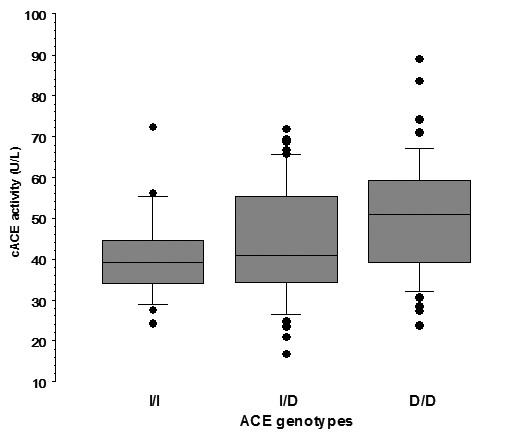
**Comparison of cACE activity results in relation with ACE genotypes**. Comparison of cACE activity results of infants with I/I (n = 18), I/D (n = 52) and D/D (n = 40) ACE genotypes. Box and whisker plot: fine horizontal lines represent 10^th^, 25^th^, 50^th^, 75^th ^and 90^th ^centiles of cACE. Outliers are plotted as discrete data points.

Eighteen infants (16.4%) were homozygous for the I-allele (I/I), 40 (36.4%) homozygous for the D-allele (D/D) and 52 infants (47.2%) were heterozygous I/D. ACE genotype distribution was in Hardy-Weinberg equilibrium (HWE), (Chi square for HWE 0.025, p = 0.874). Overall, there were no significant differences in the characteristics of the infants with I/I, D/D and I/D ACE genotypes (table 2). Neither Pi_max _measurements, nor Pi_max _adjusted for weight at measurement, were statistically different between the three groups, (table 2). Infants with D/D genotype had higher serum ACE activity than infants with I/I or I/D genotypes (Kruskal-Wallis, p = 0.028; Dunn's test, z-value = 2.37, p < 0.05 and z-value = 2.12, p < 0.05, respectively) (table 2), (figure 1). No difference, in regards to serum ACE activity, was found between infants with I/I and I/D genotypes (Dunn's test, z-value = 0.83, n.s). Linear regression analysis demonstrated that cACE activity was significantly related to Pi_max _after logarithmic transformation (r = 0.253, t-value = 2.72, p = 0.0075) and inversely related to PTImus (r = -0.238, t-value = -2.55, p = 0.012). Furthermore, stepwise regression analysis revealed that Pi_max _after logarithmic transformation was significantly related to cACE activity (p = 0.0045) and weight at measurement (p = 0.0081), independent of ACE genotyping, PMA, gender and support from mechanical ventilation (table 3). In addition, PTImus was related (inversely) to cACE activity (p = 0.00037) and to ACE genotypes (p = 0.00163), independent of weight at measurement, PMA, gender and support from mechanical ventilation (table 4).

**Table 2:** Characteristics of infants in relation to ACE genotypes

				p value
**ACE genotypes (n)**	**II (18)**	**ID (52)**	**DD (40)**	

Gestational age (weeks)	36 (27-40)	35 (28-40)	36(29-40)	0.680^a^

Birth weight (g)	2780 (960-4150)	2705 (1065-3650)	2755 (1170-3820)	0.756^a^

Male gender	9 (50.0%)	28 (53.8%)	25 (62.5%)	0.097^b^

Duration of mechanical ventilation (days)	0 (0-59)	2 (0-21)	0 (0-8)	0.167^a^

**At the time of measurement**				

Postnatal age (days)	8.5 (1-107)	9.5 (1-62)	8 (1-54)	0.686^a^

Weight (kg)	2660 (1850-4050)	2620 (1850-3680)	2657 (1880-3540)	0.618^a^

cACE activity (U/L)	39.3 (24.3-72.3)	41.1 (16.7-71.9)	51.1 (23.9-89.0)	0.028^a^

Pi_max _(cmH_2_O)	71.3 (49.3-88.7)	61.1 (36.9-90.8)	66.1 (42.4-91.8)	0.09^a^

Pi_max_/weight (cmH_2_O/kg)	25.3 (17.0-37.6)	23.5 (14.5-42.3)	24.5 (14.9-47.1)	0.267^a^

Data are demonstrated as n (%) or median (range).

^a^Kruskal-Wallis test^b^Cramer's V test

**Table 3:** Results of stepwise regression analysis of factors related to Pi_max _measurements

	Partial Correlation Coefficient	t Value	Significance
**Dependent variable: Pi**_**max**_			

cACE activity	0.262	2.899	0.0045

Weight at measurement	0.244	2.697	0.0081

ACE genotypes		0.317	0.752

Postmenstrual age		0.283	0.778

Gender		0.077	0.939

Duration of mechanicalventilation		1.451	0.150

**Table 4:** Results of stepwise regression analysis of factors related to PTImus measurements

	Partial Correlation Coefficient	t Value	Significance
**Dependent variable: PTImus**			

cACE activity	-0.339	-3.675	0.00037

ACE genotypes	0.298	3.233	0.00163

Weight at measurement		1.944	0.055

Postmenstrual age		0.792	0.430

Gender		1.364	0.169

Duration of mechanical ventilation		0.490	0.625

### Infants that never required ventilatory support

The characteristics of the infants that never required any form of ventilatory support (n = 60) are presented in table 5. In this subgroup, 10 infants (16.6%) were homozygous I/I, 25 (41.7%) homozygous D/D and 25 infants (41.7%) were heterozygous I/D (table 6). ACE genotype distribution was in Hardy-Weinberg equilibrium (HWE), (Chi square for HWE 0.741, p = 0.389).

**Table 5:** Characteristics of infants that never required respiratory support

Number of studied infants	60	
Gestational age (weeks)	37	(30-40)

Birth weight (g)	2845	(1260-3820)

Gender (male)	36	(60.0)

Preterm	28	(46.7)

**At the time of measurement**		

Postnatal age (days)	6.5	(1-31)

Postmenstrual age (PMA) (weeks)	37.6	(32.7-41.4)

Preterm (PMA<37 weeks)	22	(36.7)

Weight (g)	2765	(1880-3680)

cACE activity (U/L)	44.3	(16.7-89.0)

Pi_max _(cmH_2_O)	64.3	(36.9-91.8)

Pi_max_/weight (cmH_2_O/kg)	23.9	(14.5-47.1)

PTImus	0.061	(0.028-0.117)

Data are demonstrated as n (%) or median (range).

**Table 6:** Characteristics of infants that never required respiratory support in relation to ACE genotypes

				p value
**ACE genotypes (n)**	**II (10)**	**ID (25)**	**DD (25)**	

Gestational age(weeks)	36 (32-40)	37(30-40)	37(31-40)	0.992^a^

Birth weight (g)	2780 (1350-3650)	2820 (1500-3650)	2850 (1260-3820)	0.924^a^

Male gender	6 (60.0%)	14 (56.0%)	16 (64.0%)	0.075^b^

**At the time of measurement**				

Postnatal age (days)	7 (1-26)	6 (1-30)	8 (1-31)	0.834^a^

Weight (kg)	2675 (1950-3550)	2680 (1950-3680)	2830 (1880-3540)	0.951^a^

cACE activity (U/L)	40.2 (24.3-52.8)	41.4 (16.7-66.8)	51.8 (27.4-89.0)	0.046^a^

Pi_max _(cmH_2_O)	73.8 (49.3-88.7)	61.1 (36.9-88.4)	68.1 (47.4-91.8)	0.182^a^

Pi_max_/weight (cmH_2_O/kg)	24.9 (17.0-37.6)	22.5 (14.5-42.3)	24.3 (14.9-47.1)	0.347^a^

Data are demonstrated as n (%) or median (range).

^a^Kruskal-Wallis test^b^Cramer's V test

Infants with I/I, D/D and I/D ACE genotypes, did not differ in regards to their characteristics and in regards to either Pi_max _measurements or Pi_max _adjusted for weight at measurement (table 6). Linear regression analysis demonstrated that cACE activity was significantly related to Pi_max _after logarithmic transformation (r = 0.421, t-value = 3.532, p = 0.0008) and inversely related to PTImus (r = -0.289, t-value = -2.29, p = 0.025).

## Discussion

In this study a positive correlation between serum ACE activity and respiratory muscle strength, assessed by Pi_max _measurement, and a negative correlation between serum ACE activity and PTImus in infants, was demonstrated. Infants homozygous for the D-allele had higher cACE activity than infants homozygous for the I-allele and heterozygous I/D. Furthermore, cACE activity was related to Pi_max _and PTImus independent of other factors which could affect respiratory muscle function. The correlation between cACE activity and either Pi_max _or PTImus was replicated on a subpopulation of the main group, consisting of infants that never required any form of respiratory support.

Mouth pressures generated during crying efforts, could provide an index of respiratory muscle strength in awake infants [13]. The test has been previously validated in infants [11,12]. P_imax _measurement is a volitional test of respiratory muscle strength, however, the generated pressures produced during crying, are considered to be maximal [19].

Fatigue of respiratory muscles may result in an inability to maintain adequate alveolar ventilation and respiratory failure. Diaphragmatic pressure-time index (PTIdi) is a measure of the load-capacity ratio of the diaphragm. It describes the pressure-generating capacity of the diaphragm, independent of respiratory frequency or the type of load imposed on the respiratory system [13] and it is closely related to the endurance time, referred to as the point where the inspiratory muscles failed to maintain a task despite maximal effort [20]. The determination of PTIdi, however, is rather invasive, since it requires the placement of an esophageal catheter. Assessment of inspiratory muscle function by measurement of a non-invasive pressure-time index of the respiratory muscles (PTImus) was first described by Gaultier et al. [18]. In spontaneously breathing infants, an agreement between PTImus and PTIdi measurements using Bland and Altman analysis, was found [15].

It has been suggested, based primarily on observational evidence, that the D-allele of ACE polymorphism was associated with greater training-related strength gain and power-oriented performance [21]. An excess of the ACE D-allele has been found among elite sprint runners [22] and swimmers [8]. In addition, ACE genotypes in adults, were associated with strength response to muscle training and D-allele carriers experience greater strength increase than II homozygotes [9,23]. ACE genotype, however, is not associated with baseline muscle strength and size [24]. Furthermore, several studies have suggested that I-allele has been associated with superior exercise endurance, being found with increased frequency in elite distance runners [22], rowers [25], triathletes [26] and mountaineers [27]. A study in healthy Caucasian naïve to strength training, suggested that cACE activity was significantly associated with baseline muscle strength [10].

In the current study, ACE genotype was associated with cACE activity, which is in accordance with present literature [4,5]. Infants with D/D ACE genotype had increased cACE activity compared to infants either homozygous for the I-allele or heterozygous I/D. Although serum ACE activity was associated with increased respiratory muscle strength, such association was not demonstrated in regards to ACE genotypes. One explanation is that the deletion accounts for approximately 47% of the intra-individual variation in plasma ACE activity in Caucasians [5]. Furthermore, cACE activity is a continuous variable and would provide greater statistical power than a categorical variable such as ACE genotype. Similar results, however, have been demonstrated by others, where an association between ACE genotyping with pre-training muscle strength was not found [9,28]. Nevertheless, the correlation between Pi_max _and cACE activity is rather weak, as approximately only 7% of the variation in Pi_max _can be accounted for by the variation in cACE activity.

A maturational effect on P_imax _has been previously demonstrated [12]. Several factors could affect Pi_max_, such as gestational age, PMA, birthweight and weight at measurement [12]. Muscle mass increases with maturity and body growth [19] and P_imax _continues to increase outside the neonatal period [11]. Furthermore, ACE levels in infants have been reported to be higher than in adults [29], other studies, however, did not show any significant correlation of cACE activity with age [30]. In this study, respiratory muscle assessment was performed at the time of the blood collection, therefore, any maturational effect on P_imax _and variation on cACE activity was avoided. However, to examine the association of ACE genotype with respiratory muscle strength, P_imax _was also related to body weigth at the time of measurement.

The primary aim of this study was to examine the association of cACE activity with respiratory muscle strength in infants. Secondary aims were to investigate the potential association of cACE with PTImus measurements and ACE genotypes with cACE activity and respiratory muscle strength in this population. An association between ACE genotypes and PTImus in infants has been previously shown [31]. Thus, this issue was not examined in this study. However, ACE genotype was included in the stepwise regression analysis, as it is now known that it is strongly correlated with PTImus.

The association of cACE activity and respiratory muscle strength may be mediated through synthesis of angiotensin II (Ang II). Ang II could possibly act as a growth factor in cardiac muscle [32] and its effect may be mediated through Ang II type 1 (AT1) receptor [32]. Furthermore, Ang II may be necessary for optimal overload-induced skeletal muscle hypertrophy, acting at least in part via an AT(1) receptor-dependent pathway [33]. The physiological properties of a motor unit correlate with the histochemical properties of the constituent muscle fibres [34]. ACE D-allele compared to I-allele is associated with an increased percentage of fast-twitch type IIb skeletal muscle fibres [35], which produce greater force per unit of cross-sectional area [36]. Ang II may be also important in the redirection of blood flow from type I, fatigue-resistant, to type II, fast-twitch, muscle fibres [37]. Furthermore, in animal studies, Ang II infused into rat hindlimps increases the tension during tetanic stimulation [37]. Other actions of Ang II, that might explain the association between cACE activity and respiratory muscle strength, include the increased noradrenaline release from peripheral sympathetic nerve terminals and the CNS, facilitating sympathetic transmission [38,39]. Circulating ACE may also influence diaphragmatic muscle strength, through the degradation of kinins. In animal studies, bradykinin reduces the phenylephrine-induced hypertrophy of cardiomyocytes [40]. Thus, elevated cACE may influence muscle strength via this pathway.

Several factors may affect respiratory muscle function, such as nutrition [41], prolonged ventilatory support [42], drugs [43-45], as well as phosphate [46], calcium [47] and magnesium [48] blood levels. In addition, hypoxia [49] and hypercapnia [50] reduce diaphragmatic contractility in young piglets. All infants were measured prior to discharge, being free of any respiratory symptoms and on full enteral feeds, they did not receive any medication and their biochemistry blood tests were within the normal range during measurements.

This study has potentially important implications, given the availability of ACE inhibitors. A recent study has demonstrated that in patients with chronic heart failure, long-term therapy with ACE inhibitors improved respiratory muscle strength [51]. However, it was an uncontrolled observational study with a very small sample size. Maximum inspiratory pressure measurement is a volitional test, therefore, in order to assess respiratory muscle strength in subjects with chronic heart failure under therapy, other factors that would interfere with respiratory muscle function, should be taken into account. Some studies have demonstrated that ACE inhibitor treatment improves exercise capacity [52,53] and decrease long term decline in physical function in elderly adults [54]. However, all studies were observational and referred to either disable, hypertensive subjects or adults with congestive heart failure.

## Conclusions

These results suggest that a relation in cACE activity and respiratory muscle function, as assessed by measurement of Pi_max _and PTImus, may exist in infants. No association between ACE genotypes and Pi_max _measurements was found. In addition, an association of D-allele of ACE genotype with increased cACE activity in infants was demonstrated. Circulating ACE accounts for only a small proportion of the total body RAS, therefore, ACE activity in muscles may be a more important factor in regards to respiratory muscle properties. Further work is required to clarify the effect of ACE inhibitor treatment on respiratory muscle function.

## Abbreviations

ACE: angiotensin converting enzyme; Ang II: angiotensin II; cACE: circulating ACE; HWE: Hardy-Weinberg equilibrium; Pi_max_: maximum inspiratory pressure during crying; PMA: postmenstrual age; PTIdi: diaphragmatic pressure-time index; PTImus: pressure-time index of the respiratory muscles; RAS: renin-angiotensin system;

## Competing interests

The authors declare that they have no competing interests.

## Authors' contributions

GD, AA and SM conceived the study; DP and ST collected the subjects' samples and performed the study clinical measurements; DP and EFS performed the genetic studies; EFS and AO carried out the assays for cACE activity determination; AV collected subjects' clinical information; GD, DP and AV performed the data analysis and statistical analysis; GD and DP wrote the first draft of the paper to which all authors subsequently made contributions. All authors read and approved the final manuscript.
